# Radiomics Model for Frontotemporal Dementia Diagnosis Using T1-Weighted MRI

**DOI:** 10.3389/fnins.2022.828029

**Published:** 2022-06-20

**Authors:** Benedetta Tafuri, Marco Filardi, Daniele Urso, Roberto De Blasi, Giovanni Rizzo, Salvatore Nigro, Giancarlo Logroscino

**Affiliations:** ^1^Center for Neurodegenerative Diseases and the Aging Brain, University of Bari “Aldo Moro” at Pia Fondazione “Card. G. Panico”, Tricase, Italy; ^2^Department of Basic Medicine, Neuroscience, and Sense Organs, University of Bari “Aldo Moro”, Bari, Italy; ^3^Department of Neurosciences, King’s College London, Institute of Psychiatry, Psychology, and Neuroscience, London, United Kingdom; ^4^Department of Radiology, “Pia Fondazione Cardinale G. Panico”, Tricase, Italy; ^5^IRCCS Istituto delle Scienze Neurologiche di Bologna, Bologna, Italy; ^6^Institute of Nanotechnology (NANOTEC), Lecce, Italy

**Keywords:** frontotemporal dementia (FTD), primary progressive aphasia, behavioral variant frontotemporal dementia, radiomics, support vector machine

## Abstract

Radiomics has been proposed as a useful approach to extrapolate novel morphological and textural information from brain Magnetic resonance images (MRI). Radiomics analysis has shown unique potential in the diagnostic work-up and in the follow-up of patients suffering from neurodegenerative diseases. However, the potentiality of this technique in distinguishing frontotemporal dementia (FTD) subtypes has so far not been investigated. In this study, we explored the usefulness of radiomic features in differentiating FTD subtypes, namely, the behavioral variant of FTD (bvFTD), the non-fluent and/or agrammatic (PNFA) and semantic (svPPA) variants of a primary progressive aphasia (PPA). Classification analyses were performed on 3 Tesla T1-weighted images obtained from the Frontotemporal Lobar Degeneration Neuroimaging Initiative. We included 49 patients with bvFTD, 25 patients with PNFA, 34 patients with svPPA, and 60 healthy controls. Texture analyses were conducted to define the first-order statistic and textural features in cortical and subcortical brain regions. Recursive feature elimination was used to select the radiomics signature for each pairwise comparison followed by a classification framework based on a support vector machine. Finally, 10-fold cross-validation was used to assess classification performances. The radiomics-based approach successfully identified the brain regions typically involved in each FTD subtype, achieving a mean accuracy of more than 80% in distinguishing between patient groups. Note mentioning is that radiomics features extracted in the left temporal regions allowed achieving an accuracy of 91 and 94% in distinguishing patients with svPPA from those with PNFA and bvFTD, respectively. Radiomics features show excellent classification performances in distinguishing FTD subtypes, supporting the clinical usefulness of this approach in the diagnostic work-up of FTD.

## Introduction

Frontotemporal dementia (FTD) encompasses a spectrum of neurodegenerative diseases characterized by behavioral changes, language abnormalities, executive dysfunctions, and social cognition impairment (Bang et al., 2015). Current diagnostic criteria recognize three main clinical subtypes of FTD: the behavioral variant of FTD (bvFTD) (Rascovsky et al., 2011), the semantic variant of primary progressive aphasia (svPPA), and the non-fluent/agrammatic variant of PPA (PNFA) (Gorno-Tempini et al., 2011). Nonetheless, FTD diagnosis remains challenging due to the high degree of clinical overlap among FTD subtypes, especially in the early disease stage.

Therefore, several neuroimaging findings, most notably, degeneration of the frontal and/or anterior temporal brain regions, have been proposed to support the clinical diagnosis (Gorno-Tempini et al., 2011; Rascovsky et al., 2011). In this context, gray matter abnormalities evaluated using region of interest (ROI) analyses or voxel- and surface-based approaches have been proven useful in differentiating FTD subtypes (McCarthy et al., 2018). In particular, the pattern of atrophy in patients with bvFTD is characterized by prominent involvement of the frontal lobe, anterior cingulate cortex, and basal ganglia (Pan et al., 2012; Möller et al., 2016; Meyer et al., 2017; Manera et al., 2021). By contrast, PPA involves the language-dominant hemisphere primarily associated with left-sided atrophy, more specifically PNFA with inferior frontal and insular atrophy, and svPPA with anterior temporal atrophy (Wilson et al., 2009; Agosta et al., 2015; Bisenius et al., 2017; Kim et al., 2019).

In recent years, radiomics has been proposed as a useful method to extrapolate novel morphological and textural features from imaging data. This approach assesses the interrelationships between image pixel gray levels and patterns, providing second-order statistics able to capture integrity properties at the microstructural level. Radiomics and machine learning approaches have been successfully implemented in oncology to differentiate pathological tissues by examining multimodality imaging (Gillies et al., 2016). More recently, radiomics has been applied to support the clinical classification of patients with neurodegenerative disorders (Feng et al., 2018; Ranjbar et al., 2019; Salvatore et al., 2019). More in detail, radiomics features of the hippocampus and corpus callosum have been used to distinguish between patients with Alzheimer’s disease and mild cognitive impairment (Feng et al., 2018; Ranjbar et al., 2019) and to differentiate patients with Parkinson’s disease from healthy controls (Cao et al., 2020). Nonetheless, studies assessing the potentiality of the radiomics approach to distinguishing FTD subtypes are lacking.

In the present study, we aimed to investigate whether radiomics features, evaluated on T1-weighted Magnetic resonance images (MRI) images, could support the clinical differentiation of FTD subtypes. In particular, texture features were extracted in cortical and subcortical gray matter regions using 1st-order and 2nd-order statistics methods. Then, a classification framework based on a support vector machine approach was applied to distinguish FTD subtypes.

## Materials and Methods

### Patients

Data used in the preparation of this retrospective study were obtained from the Frontotemporal Lobar Degeneration Neuroimaging Initiative (FTLDNI) database (for up-to-date information on participation and protocol).^1^ We considered 60 healthy control (HC) and 108 patients with FTD (49 bvFTD, 25 PNFA, and 34 svPPA), who had valid baseline T1-weighted MR images. To avoid potential bias derived from different imaging protocols, we selected exclusively images acquired at the University of California, San Francisco (UCSF), i.e., the largest recruiting center.

All the patients underwent comprehensive neurological, neuropsychological, and functional assessments and were diagnosed according to the current diagnostic criteria (Gorno-Tempini et al., 2011; Rascovsky et al., 2011). The individuals with no previous history of diagnosed neurological or psychiatric disorder and no complaint of memory deterioration (more information)^2^ served as the control group.

### Magnetic Resonance Images Data Acquisition and Preprocessing

Magnetic resonance images were acquired on a 3T Siemens Trio Tim system equipped with a 12-channel head coil at the UCSF Neuroscience Imaging Center, including whole-brain three-dimensional T1 MPRAGE (TR/TE = 2,300/2.9 ms, matrix = 240 × 256 × 160, isotropic voxels 1 mm^3^, slice thickness = 1 mm). An experienced neuroradiologist reviewed the images for brain abnormalities other than atrophy.

For each subject, T1-weighted images were converted from DICOM to NiFTI (NeuroInformatics Technology Initiative). Cortical and subcortical regions were segmented using the recon-all script included in Freesurfer v6.0.^3^ In particular, we defined 34 cortical regions of interest (ROIs) per hemisphere by using the Desikan-Killiany atlas cortical parcellation (Desikan et al., 2006). Seven subcortical ROIs (Thalamus, Caudate, Putamen, Amygdala, Hippocampus, Pallidum, and Accumbens) were also defined (Fischl et al., 2002). Each region was used as a mask to extract radiomic features. Segmentation results were also visually inspected by an expert neuroradiologist (R.D.B.), and no manual edits were necessary.

### Radiomics Features Computation

As a first step, minimal preprocessing (i.e., standardizing the grayscale levels) was performed to harmonize the data. Subsequently, we computed radiomics features from each cortical and subcortical brain region through the PyRadiomics software (van Griethuysen et al., 2017). We computed first-order statistic features, describing the distribution of voxel intensities within the ROI mask, and, to quantify intra-ROI heterogeneity, we extracted textural features by analyzing the gray-level co-occurrence matrix, run-length matrix, dependence matrix, and size-zone matrix (Zwanenburg et al., 2020). Detailed information can be found in Supplementary Table 1.

### Machine-Learning-Based Analysis

In our framework, we considered all binary comparisons between groups. For each initial dataset, we handled possible imbalanced learning problems using a Majority Weighted Minority Oversampling Technique (MWMOTE) that allows us to generate additional samples for minority classes (Barua et al., 2014). Thereafter, each model was evaluated in a 10-fold cross-validation setting, including feature selection and classification steps.

To construct a radiomics signature, we used a dimensionality reduction algorithm, namely, the Random-Forest-Recursive Feature Elimination (RF-RFE) (Gregorutti et al., 2017). This supervised method iteratively trains the model, ranks features, and retains a subset of the most relevant descriptors to produce an accurate model. This procedure was implemented with a repeated 10-fold cross-validation. The final number of selected features followed the criterion proposed by Hua et al. (2005), which suggests an optimal size proportional to √N (where N is the sample size) for high-correlated features. In our models, we retained a number of features ≤√N in accordance with the optimal accuracy values achieved.

As a second step, to assess the prediction performance of radiomics signatures, we defined a machine learning model based on a Support Vector Machine (SVM) classifier using the libSVM package (Chang and Lin, 2011). In the training step, the most informative features extracted in the first step were used to construct the SVM model by a radial basis kernel. We tuned hyperparameters using a 10-fold cross-validation approach on the training sets, namely, we adopted a grid search method to optimize the two parameters of SVM, such as *c*, the width of the RBF, and *C*, an input parameter for the SVM algorithm, which controls the trade-off between having zero training errors and allowing misclassification. We minimized the classification error, searching the “best model” for (c, C), varying along a grid with *c* = 0.1, 0.5, 1, 2, 3, 4, and *C* = 0.001, 0.01, 0.1, 1, 5, 10. In the testing step, we retained only descriptors selected by RF-RFE and evaluated the performance of the pre-trained SVM model. Finally, we evaluated the feature frequencies and the mean performances for each cross-validated model.

### Statistical Analysis

Data were explored with descriptive statistics (mean ± SD). Group differences in age, sex, education, MMSE (Mini-Mental State Examination), and CDR (Clinical Dementia Rating Scale) scores were investigated through the Chi-square test, one-way ANOVA, and Kruskal–Wallis ANOVA, followed by *post hoc* comparisons. For all analyses, the corrected significance threshold was set at *p* < 0.05 after Bonferroni correction for multiple comparisons. Statistical analysis was performed by using R software (Version 3.6.3: R Foundation for Statistical Computing, Vienna, Austria).

Classification performances were evaluated by accuracy, sensitivity, and specificity. Finally, the diagnostic capabilities of the radiomics signatures were evaluated with Receiver Operating Characteristic (ROC) curve analysis.

## Results

### Demographic and Clinical Data

Demographic and clinical data are reported in Table 1. No significant differences emerged in age and sex distribution. The patients with svPPA showed lower years of education than controls (*p* < 0.001, Bonferroni corrected). Concerning clinical data, all the patient groups had significantly lower MMSE and CDR scores than the control (*p*-value < 0.001, Bonferroni corrected).

**TABLE 1:** Patient demographics.

	HC (*n* = 60)	bvFTD (*n* = 49)	PNFA (*n* = 25)	svPPA (*n* = 34)	*P*-value
	Mean ± SD	Mean ± SD	Mean ± SD	Mean ± SD	
Age, y	64.3 ± 4.9	61.3 ± 6.9	65.2 ± 5.6	62.9 ± 6.3	Ns
Sex (male%)	0.58	0.62	0.56	0.44	Ns
Education, y	17.5 ± 1.9	15.4 ± 3.3	15.6 ± 2.6	13.9 ± 8.4	0.005*
MMSE	29.4 ± 0.7	23.6 ± 4.5	25.4 ± 4.3	24.9 ± 5.1	<0.001**
CDR	0.0 ± 0.1	1.2 ± 0.6	0.5 ± 0.4	0.6 ± 0.3	<0.001**

*HC vs. svPPA, p = 0.001.

**HC vs. bvFTD, PNFA, svPPA, p < 0.001.

*HC, healthy controls; bvFTD, behavioral variant frontotemporal dementia; PNFA, non-fluent/agrammatic variant of primary progressive aphasia; svPPA, semantic variant of primary progressive aphasia; MMSE, Mini-Mental State Examination; CDR, Clinical Dementia Rating Scale.*

### Classification Analysis

**FIGURE 1:**
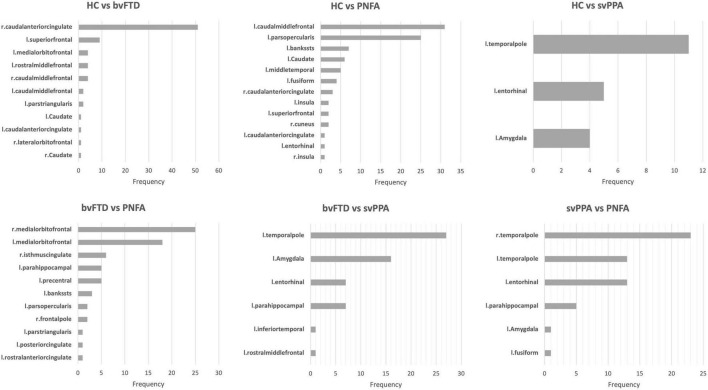
The frequency of extracted ROIs for each binary model.

To define which radiomics predictors were most influential in the differentiation of the considered groups, we studied the frequency of the features selected in the 10-fold cross-validation (see Supplementary Table 2). The frequency of ROIs with significative radiomic measures for each binary model is reported in Figure 1. Compared to healthy controls, patients with bvFTD showed a bilateral distribution of involved regions with a predominance of frontal and caudal anterior cingulate cortices. On the other hand, PNFA and svPPA subtypes showed a more left-sided pattern of ROIs. In particular, the patients affected by PNFA differed from the control group in the left caudal middle frontal gyrus, left pars opercularis, left middle temporal gyrus, and caudate. The patients with svPPA showed a temporal compromission of the left hemisphere, comprising the temporal pole, entorhinal and amygdalar regions. Comparisons of bvFTD with PNFA subjects showed a predominance of features in the medial orbitofrontal cortex bilaterally, while the comparison with svPPA reported a similar left temporal pattern as for controls. Lastly, the involvement of the temporal pole, entorhinal, and parahippocampus was highlighted in the differentiation between svPPA and PNFA. Note mentioning is the left temporal pole features have been selected in radiomic signatures for comparison with HC, bvFTD, and PNFA models thus representing an optimal discriminative characteristic of an svPPA subtype.

Mean classification performances for all trained models are reported in Table 2. With respect to healthy controls, patients with bvFTD were correctly classified with an 85% of accuracy (sensitivity: 84%; specificity: 88%), patients with PNFA with an 84% accuracy (sensitivity: 91%; specificity: 82%) and patients with svPPA, with a 98% accuracy (sensitivity: 98%; specificity: 98%). Classification models between bvFTD and PPA subtypes reported an accuracy value of 80 and 94%, respectively, for patients with PNFA and patients with svPPA. Lastly, svPPA and PNFA patient comparison reported performance of 91% accuracy (sensitivity: 88%; specificity: 93%).

**TABLE 2:** Evaluation metrics (mean ± SD) of the binary models computed with the 10-fold cross- validation.

	Accuracy (mean ± SD)	Sensitivity (mean ± SD)	Specificity (mean ± SD)
HC vs. bvFTD	0.85 ± 0.09	0.84 ± 0.16	0.88 ± 0.13
HC vs. PNFA	0.84 ± 0.08	0.91 ± 0.10	0.82 ± 0.15
HC vs. svPPA	0.98 ± 0.04	0.98 ± 0.06	0.98 ± 0.06
bvFTD vs. PNFA	0.80 ± 0.15	0.90 ± 0.17	0.75 ± 0.18
bvFTD vs. svPPA	0.94 ± 0.10	0.90 ± 0.21	0.95 ± 0.11
svPPA vs. PNFA	0.91 ± 0.07	0.88 ± 0.16	0.93 ± 0.14

*HC, healthy controls; bvFTD, behavioral variant frontotemporal dementia; PNFA, non-fluent/agrammatic variant of primary progressive aphasia; svPPA, semantic variant of primary progressive aphasia.*

**FIGURE 2:**
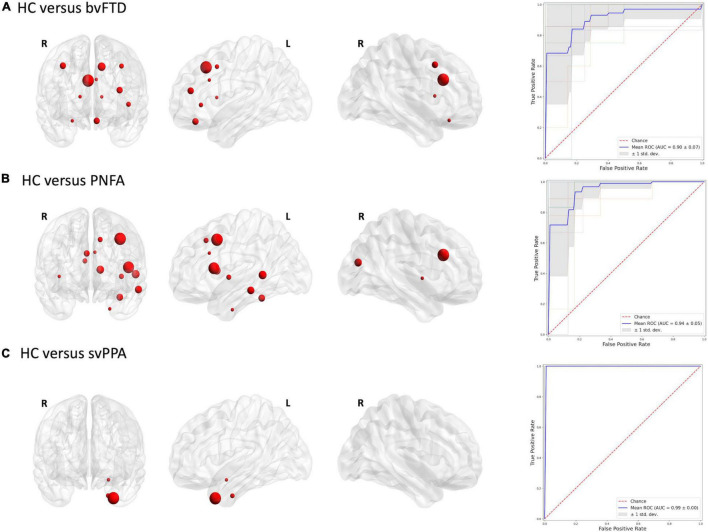
Discriminative regions in the radiomics approach and ROC curves. We report extracted ROI from subjects’ comparison **(A)** HC vs. bvFTD; **(B)** HC vs. PNFA; **(C)** HC vs. svPPA. HC, healthy controls; bvFTD, behavioral variant frontotemporal dementia; PNFA, non-fluent/agrammatic variant of primary progressive aphasia; svPPA, semantic variant of primary progressive aphasia.

**FIGURE 3:**
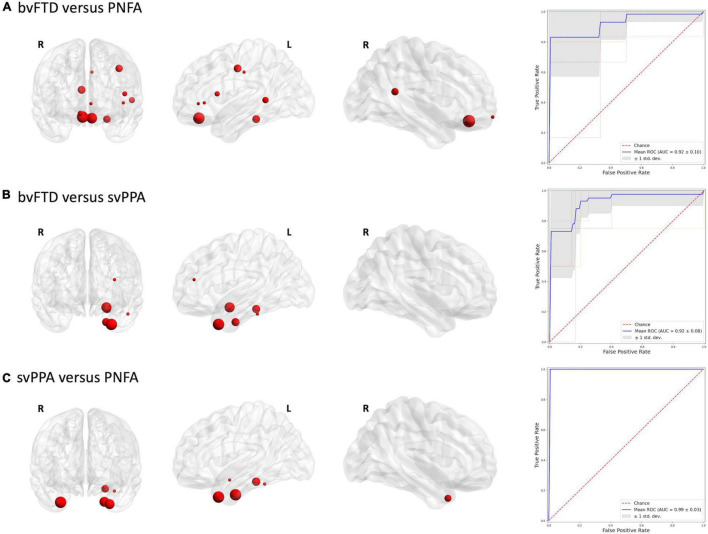
Discriminative regions in the radiomics approach and ROC curves. We report extracted ROI from subjects’ comparison **(A)** bvFTD vs. PNFA; **(B)** bvFTD vs. svPPA; **(C)** svPPA vs. PNFA. HC, healthy controls; bvFTD, behavioral variant frontotemporal dementia; PNFA, non-fluent/agrammatic variant of primary progressive aphasia; svPPA, semantic variant of primary progressive aphasia.

Figures 2, 3 report the discriminative regions selected for each model, the corresponding ROC curves, and the relative AUCs.

## Discussion

In this study, we analyzed, for the first time, the utility of the radiomics approach in the diagnostic work-up of FTD subtypes. Overall, radiomics features were able to classify FTD subtypes from healthy controls with optimal accuracy. Similar results were also observed in distinguishing between patient groups. Of note, an excellent accuracy was found in differentiating patients with svPPA from those with bvFTD or PNFA.

The lack of radiomics studies in patients with FTD spectrum precludes the possibility of directly compare our findings with existing literature. Nonetheless, our findings are in line with previous studies that adopted voxel-based or surface-based morphometry approaches. Indeed, the areas identified through radiomics overlapped with the typically observed compromission networks (McCarthy et al., 2018). Binary classification results for bvFTD vs. controls confirmed the crucial role of the caudal anterior cingulate cortex and superior frontal gyrus in the pathophysiological process of the bvFTD (Pan et al., 2012; Gordon et al., 2016; Möller et al., 2016; Meyer et al., 2017; Manera et al., 2021; Nigro et al., 2021b). On the other hand, morphometric studies conducted on PPA variants found a predominantly left pattern of atrophy, associated with language deficit, and demonstrated a good discriminative power (Wilson et al., 2009; Agosta et al., 2015; Bisenius et al., 2017; Kim et al., 2019). Consistent with these studies, our study showed a prominent involvement of the caudal middle frontal gyrus, pars opercularis regions and caudate for PNFA vs. the controls classification model. Similarly, predominant involvement of temporal brain regions, i.e., temporal pole, hippocampus, and amygdala, was also observed for the classification between svPPA and control (Gorno-Tempini et al., 2004; Rohrer et al., 2009b; Bocchetta et al., 2019; Nigro et al., 2021a).

Concerning FTD subtype comparisons, the behavioral variant of FTD highlighted a prefrontal and temporoparietal network, more shifted on the right hemisphere, when compared with PNFA (Wilson et al., 2010). By contrast, a specific pattern of radiomics features in left temporal regions with predominance in the temporal pole was found in differentiating patients with bvFTD from patients with svPPA (Bruun et al., 2019). Moreover, radiomics features extracted in the left temporal pole allowed to distinguish patients with svPPA from those with PNFA, confirming previous evidences of the crucial role of this region in the neurodegenerative mechanisms underlying svPPA phenotype (Collins et al., 2017). Concerning classification performances, our findings overcome the performance described in previous studies using morphometric properties, such as cortical thickness to distinguish FTD subtypes (Wilson et al., 2009; Agosta et al., 2015; Bisenius et al., 2017; Bruun et al., 2019; Kim et al., 2019). Indeed, our radiomics model between patients with svPPA and patients with bvFTD outclassed the existent model based on the brain atrophy indexes in terms of better sensitivity and specificity from 80 and 93% to 90 and 95%, respectively (Bruun et al., 2019). Note mentioning is that radiomics features achieved a diagnostic accuracy of 91% in differentiating patients with svPPA from patients with PNFA, highlighting the usefulness of this approach in providing unique information associated with these diseases.

Apart from the classification task, radiomics could display unique advantages in the field of frontotemporal dementia. Radiomics features have, in fact, demonstrated an optimal predictive power in terms of response to therapy or clinical outcomes in the field of oncology and neurodegeneration (Feng and Ding, 2020; Conti et al., 2021; Kim et al., 2021). Hence, radiomics, even in combination with non-imaging data such as clinical scales and biological markers, might reasonably be used to enhance the predictive potential of medical imaging in FTD subtypes. Indeed, recent investigations in the oncology field have demonstrated that the combination of radiomics and genomic data can represent a turning point in the field of precision medicine by facilitating computer-aided diagnosis, treatment, and prediction of the prognosis (Shui et al., 2020; Bera et al., 2021). Bearing in mind the abovementioned studies and considering that up to 20–40% of FTD cases have a family history of the disease (Rohrer et al., 2009a), the approach of combining radiomics and genomics features may show unique potential not only in FTD clinical work-up but also as possible presymptomatic signature (Feis et al., 2019, 2020; Premi et al., 2022). Indeed, previous studies have shown that machine-learning applied to structural MRI data has proved a reliable method to track neuroanatomical changes of patients with FTD at a single-subject level and has been proposed as an early diagnostic marker for presymptomatic Granulin mutation carriers.

Some limitations of the present study should be acknowledged. First, we lack a validation cohort, acquired with a different scan protocol, to test the generalizability of our models and to improve the statistical power of the results. Secondly, although the number of the participants is comparable with previous classification studies, the sample size should be increased to define a more generalizable radiomics signature of FTD subtypes. Further studies should also be conducted to investigate the classification performance of radiomics features when combined with clinical and neuropsychological data. Finally, multimodal MRI analyses, combining textural radiomics features with diffusional and functional properties, could achieve a powerful diagnostic tool for clinical application.

To conclude, our study showed, for the first time, the usefulness of the radiomic approach in the diagnostic work-up of FTD. Radiomics features could be considered in clinical practice as a reliable and practical marker to identify patients in the frontotemporal dementia spectrum and, potentially, an important predictor of treatment response.

## Data Availability Statement

The raw data supporting the conclusions of this article will be made available by the corresponding author, upon reasonable request.

## Ethics Statement

Ethical review and approval were not required for the study on human participants in accordance with the local legislation and institutional requirements. Written informed consent for participation was not required for this study in accordance with the national legislation and the institutional requirements.

## Author Contributions

BT, SN, and GL contributed to conception and design of the study. BT, SN, MF, DU, RD, and GR contributed to analysis and interpretation of data. BT drafted the article. SN, DU, MF, RD, GR, and GL contributed to revise it critically for important intellectual content. BT, SN, MF, DU, RD, GR, and GL provided approval for publication of the version to be published and agreed to be accountable for all aspects of the work in ensuring that questions related to the accuracy or integrity of any part of the work are appropriately investigated and resolved. All authors contributed to the article and approved the submitted version.

## Conflict of Interest

The authors declare that the research was conducted in the absence of any commercial or financial relationships that could be construed as a potential conflict of interest.

## Publisher’s Note

All claims expressed in this article are solely those of the authors and do not necessarily represent those of their affiliated organizations, or those of the publisher, the editors and the reviewers. Any product that may be evaluated in this article, or claim that may be made by its manufacturer, is not guaranteed or endorsed by the publisher.

## Footnotes

1
http://memory.ucsf.edu/research

2
https://memory.ucsf.edu/research-trials/research/4rtni-2

3
https://surfer.nmr.mgh.harvard.edu/
