# Supplementary material for: Radiomics Model for Frontotemporal Dementia Diagnosis Using T1-Weighted MRI
Source: Front Neurosci. 2022 Jun 20;16:828029. doi: 10.3389/fnins.2022.828029 (PMC9251132; doi:10.3389/fnins.2022.828029)
Supplement: Supplementary file 1 [file Data_Sheet_1.docx]

**Radiomics model for frontotemporal dementia diagnosis using T1-weighted MRI**

**Supplementary materials**

**Supplementary Table 1.** Detailed features extracted from each region-of-interest.

| Type | Measure |
| --- | --- |
| First order | *Interquartile Range* |
|  | *Skewness* |
|  | *Uniformity* |
|  | *Median* |
|  | *Energy* |
|  | *Robust Mean Absolute Deviation* |
|  | *Total Energy* |
|  | *Maximum* |
|  | *Root Mean Squared* |
|  | *90° Percentile* |
|  | *Minimum* |
|  | *Entropy* |
|  | *Range* |
|  | *Variance* |
|  | *10° Percentile* |
|  | *Kurtosis* |
|  | *Mean* |
|  | *Mean Absolute Deviation* |
| GLDM | *Gray Level Variance* |
| (Gray Level Dependence matrix) | *High Gray Level Emphasis* |
|  | *Dependence Entropy* |
|  | *Dependence Non Uniformity* |
|  | *Gray Level Non Uniformity* |
|  | *Small Dependence Emphasis* |
|  | *Small Dependence High Gray Level Emphasis* |
|  | *Dependence Non Uniformity Normalized* |
|  | *Large Dependence Emphasis* |
|  | *Large Dependence Low Gray Level Emphasis* |
|  | *Dependence Variance* |
|  | *Large Dependence High Gray Level Emphasis* |
|  | *Small Dependence Low Gray Level Emphasis* |
|  | *Low Gray Level Emphasis* |
| GLCM | *Joint Average* |
| (Gray level Cooccurence matrix) | *Sum Average* |
|  | *Joint Entropy* |
|  | *Cluster Shade* |
|  | *Maximum Probability* |
|  | *Inverse Difference Moment Normalized* |
|  | *Joint Energy* |
|  | *Contrast* |
|  | *Difference Entropy* |
|  | *Inverse Variance* |
|  | *Difference Variance* |
|  | *Inverse Difference Normalized* |
|  | *Inverse Difference Moment* |
|  | *Correlation* |
|  | *Autocorrelation* |
|  | *Sum Entropy* |
|  | *Maximal Correlation Coefficient* |
|  | *Sum Squared* |
|  | *Cluster Prominence* |
|  | *Informational Measure of Correlation 2* |
|  | *Informational Measure of Correlation 1* |
|  | *Difference Average* |
|  | *Inverse Difference* |
|  | *Cluster Tendency* |
| GLRLM | *Short Run Low Gray Level Emphasis* |
| (gray level run length matrix) | *Gray Level Variance* |
|  | *Low Gray Level Non Uniformity Normalized* |
|  | *Gray Level Non Uniformity Normalized* |
|  | *Run Variance* |
|  | *Gray Level Non Uniformity* |
|  | *Long Run Emphasis* |
|  | *Short Run High Gray Level Emphasis* |
|  | *Run Length Non Uniformity* |
|  | *Short Run Non Uniformity* |
|  | *Long Run High Gray Level Emphasis* |
|  | *Run Percentage* |
|  | *Long Run Low Gray Level Emphasis* |
|  | *Run Entropy* |
|  | *High Gray Level Run Emphasis* |
|  | *Run Length Non Uniformity Normalized* |
| GLSZM | *Gray Level Variance* |
| (gray level size zone matrix) | *Zone Variance* |
|  | *Gray Level Non Uniformity Normalized* |
|  | *Size Zone Non Uniformity Normalized* |
|  | *Size Zone Non Uniformity* |
|  | *Gray Level Non Uniformity* |
|  | *Large Area Emphasis* |
|  | *Small Area High Gray Level Emphasis* |
|  | *Zone Percentage* |
|  | *Large Area Low Gray Level Emphasis* |
|  | *Large Area High Gray Level Emphasis* |
|  | *High Gray Level Zone Emphasis* |
|  | *Small Area Emphasis* |
|  | *Low Gray Level Zone Emphasis* |
|  | *Zone Entropy* |
|  | *Small Area Low Gray Level Emphasis* |

**Supplementary Table 2.** Radiomics features selected for each model and region of interest.

| **Binary Model** | **Selected feature** | **Region of Interest** | **Frequency** |
| --- | --- | --- | --- |
| bvFTD_PNFA | glcm_DifferenceEntropy | rh-medialorbitofrontal | 6 |
| bvFTD_PNFA | glrlm_RunLengthNonUniformityNormalized | rh-medialorbitofrontal | 5 |
| bvFTD_PNFA | glrlm_ShortRunEmphasis | rh-medialorbitofrontal | 4 |
| bvFTD_PNFA | glcm_Contrast | rh-medialorbitofrontal | 3 |
| bvFTD_PNFA | gldm_SmallDependenceEmphasis | rh-medialorbitofrontal | 3 |
| bvFTD_PNFA | glcm_DifferenceVariance | rh-medialorbitofrontal | 1 |
| bvFTD_PNFA | glcm_InverseVariance | rh-medialorbitofrontal | 1 |
| bvFTD_PNFA | gldm_LargeDependenceLowGrayLevelEmphasis | rh-medialorbitofrontal | 1 |
| bvFTD_PNFA | glrlm_RunPercentage | rh-medialorbitofrontal | 1 |
| bvFTD_PNFA | glszm_LargeAreaHighGrayLevelEmphasis | rh-isthmuscingulate | 6 |
| bvFTD_PNFA | glcm_DifferenceAverage | rh-frontalpole | 1 |
| bvFTD_PNFA | glrlm_RunLengthNonUniformityNormalized | rh-frontalpole | 1 |
| bvFTD_PNFA | gldm_LargeDependenceLowGrayLevelEmphasis | lh-rostralanteriorcingulate | 1 |
| bvFTD_PNFA | gldm_DependenceNonUniformity | lh-precentral | 5 |
| bvFTD_PNFA | glcm_ClusterShade | lh-posteriorcingulate | 1 |
| bvFTD_PNFA | glszm_GrayLevelNonUniformity | lh-parstriangularis | 1 |
| bvFTD_PNFA | gldm_DependenceNonUniformity | lh-parsopercularis | 2 |
| bvFTD_PNFA | glszm_SizeZoneNonUniformityNormalized | lh-parahippocampal | 2 |
| bvFTD_PNFA | glszm_SmallAreaEmphasis | lh-parahippocampal | 2 |
| bvFTD_PNFA | firstorder_Range | lh-parahippocampal | 1 |
| bvFTD_PNFA | gldm_LargeDependenceLowGrayLevelEmphasis | lh-medialorbitofrontal | 6 |
| bvFTD_PNFA | glrlm_ShortRunEmphasis | lh-medialorbitofrontal | 3 |
| bvFTD_PNFA | glszm_LargeAreaLowGrayLevelEmphasis | lh-medialorbitofrontal | 3 |
| bvFTD_PNFA | gldm_SmallDependenceHighGrayLevelEmphasis | lh-medialorbitofrontal | 2 |
| bvFTD_PNFA | glrlm_RunLengthNonUniformityNormalized | lh-medialorbitofrontal | 2 |
| bvFTD_PNFA | glrlm_RunPercentage | lh-medialorbitofrontal | 2 |
| bvFTD_PNFA | glcm_InverseVariance | lh-bankssts | 3 |
| bvFTD_svPPA | glrlm_RunLengthNonUniformity | lh-temporalpole | 7 |
| bvFTD_svPPA | firstorder_Skewness | lh-temporalpole | 6 |
| bvFTD_svPPA | gldm_DependenceNonUniformity | lh-temporalpole | 5 |
| bvFTD_svPPA | firstorder_Energy | lh-temporalpole | 3 |
| bvFTD_svPPA | firstorder_TotalEnergy | lh-temporalpole | 3 |
| bvFTD_svPPA | glcm_ClusterShade | lh-temporalpole | 3 |
| bvFTD_svPPA | glszm_ZonePercentage | lh-rostralmiddlefrontal | 1 |
| bvFTD_svPPA | firstorder_Energy | lh-parahippocampal | 4 |
| bvFTD_svPPA | firstorder_TotalEnergy | lh-parahippocampal | 3 |
| bvFTD_svPPA | glrlm_RunLengthNonUniformity | lh-inferiortemporal | 1 |
| bvFTD_svPPA | firstorder_Energy | lh-entorhinal | 3 |
| bvFTD_svPPA | glrlm_RunLengthNonUniformity | lh-entorhinal | 3 |
| bvFTD_svPPA | firstorder_TotalEnergy | lh-entorhinal | 1 |
| bvFTD_svPPA | firstorder_Energy | Left-Amygdala | 8 |
| bvFTD_svPPA | firstorder_TotalEnergy | Left-Amygdala | 8 |
| HC_bvFTD | glszm_LargeAreaEmphasis | Right-Caudate | 1 |
| HC_bvFTD | firstorder_Skewness | rh-lateralorbitofrontal | 1 |
| HC_bvFTD | glszm_ZonePercentage | rh-caudalmiddlefrontal | 4 |
| HC_bvFTD | firstorder_Variance | rh-caudalanteriorcingulate | 9 |
| HC_bvFTD | firstorder_Entropy | rh-caudalanteriorcingulate | 8 |
| HC_bvFTD | gldm_GrayLevelVariance | rh-caudalanteriorcingulate | 7 |
| HC_bvFTD | glrlm_GrayLevelVariance | rh-caudalanteriorcingulate | 7 |
| HC_bvFTD | glrlm_RunEntropy | rh-caudalanteriorcingulate | 7 |
| HC_bvFTD | glszm_GrayLevelVariance | rh-caudalanteriorcingulate | 6 |
| HC_bvFTD | glszm_GrayLevelNonUniformityNormalized | rh-caudalanteriorcingulate | 4 |
| HC_bvFTD | gldm_DependenceEntropy | rh-caudalanteriorcingulate | 2 |
| HC_bvFTD | glcm_ClusterProminence | rh-caudalanteriorcingulate | 1 |
| HC_bvFTD | gldm_SmallDependenceEmphasis | lh-superiorfrontal | 5 |
| HC_bvFTD | glszm_ZonePercentage | lh-superiorfrontal | 4 |
| HC_bvFTD | gldm_SmallDependenceEmphasis | lh-rostralmiddlefrontal | 2 |
| HC_bvFTD | glszm_ZonePercentage | lh-rostralmiddlefrontal | 2 |
| HC_bvFTD | glcm_ClusterShade | lh-parstriangularis | 1 |
| HC_bvFTD | glcm_ClusterShade | lh-parsopercularis | 1 |
| HC_bvFTD | firstorder_Skewness | lh-medialorbitofrontal | 4 |
| HC_bvFTD | gldm_SmallDependenceEmphasis | lh-caudalmiddlefrontal | 1 |
| HC_bvFTD | glszm_ZonePercentage | lh-caudalmiddlefrontal | 1 |
| HC_bvFTD | glcm_Contrast | lh-caudalanteriorcingulate | 1 |
| HC_bvFTD | glszm_ZoneVariance | Left-Caudate | 1 |
| HC_PNFA | glcm_ClusterShade | rh-insula | 1 |
| HC_PNFA | glcm_Imc1 | rh-cuneus | 2 |
| HC_PNFA | glrlm_RunEntropy | rh-caudalanteriorcingulate | 3 |
| HC_PNFA | glszm_ZonePercentage | lh-superiorfrontal | 2 |
| HC_PNFA | glcm_ClusterShade | lh-parsopercularis | 10 |
| HC_PNFA | gldm_GrayLevelNonUniformity | lh-parsopercularis | 7 |
| HC_PNFA | glrlm_GrayLevelNonUniformity | lh-parsopercularis | 6 |
| HC_PNFA | glcm_ClusterProminence | lh-parsopercularis | 2 |
| HC_PNFA | glcm_ClusterShade | lh-middletemporal | 3 |
| HC_PNFA | glszm_LargeAreaLowGrayLevelEmphasis | lh-middletemporal | 2 |
| HC_PNFA | glcm_ClusterShade | lh-insula | 2 |
| HC_PNFA | glcm_ClusterShade | lh-fusiform | 4 |
| HC_PNFA | glcm_DifferenceVariance | lh-entorhinal | 1 |
| HC_PNFA | glcm_ClusterShade | lh-caudalmiddlefrontal | 7 |
| HC_PNFA | gldm_SmallDependenceEmphasis | lh-caudalmiddlefrontal | 6 |
| HC_PNFA | glszm_ZonePercentage | lh-caudalmiddlefrontal | 6 |
| HC_PNFA | glcm_DifferenceVariance | lh-caudalmiddlefrontal | 4 |
| HC_PNFA | glcm_ClusterProminence | lh-caudalmiddlefrontal | 2 |
| HC_PNFA | glrlm_GrayLevelVariance | lh-caudalmiddlefrontal | 2 |
| HC_PNFA | glszm_LargeAreaLowGrayLevelEmphasis | lh-caudalmiddlefrontal | 2 |
| HC_PNFA | gldm_GrayLevelNonUniformity | lh-caudalmiddlefrontal | 1 |
| HC_PNFA | gldm_GrayLevelVariance | lh-caudalmiddlefrontal | 1 |
| HC_PNFA | firstorder_Maximum | lh-caudalanteriorcingulate | 1 |
| HC_PNFA | glcm_ClusterShade | lh-bankssts | 5 |
| HC_PNFA | firstorder_Skewness | lh-bankssts | 1 |
| HC_PNFA | glszm_LargeAreaLowGrayLevelEmphasis | lh-bankssts | 1 |
| HC_PNFA | glszm_ZonePercentage | Left-Caudate | 4 |
| HC_PNFA | glszm_LargeAreaEmphasis | Left-Caudate | 1 |
| HC_PNFA | glszm_ZoneVariance | Left-Caudate | 1 |
| HC_svPPA | glrlm_RunLengthNonUniformity | lh-temporalpole | 4 |
| HC_svPPA | firstorder_TotalEnergy | lh-temporalpole | 2 |
| HC_svPPA | glcm_ClusterShade | lh-temporalpole | 2 |
| HC_svPPA | firstorder_Energy | lh-temporalpole | 1 |
| HC_svPPA | firstorder_Skewness | lh-temporalpole | 1 |
| HC_svPPA | glrlm_GrayLevelNonUniformity | lh-temporalpole | 1 |
| HC_svPPA | firstorder_Energy | lh-entorhinal | 1 |
| HC_svPPA | firstorder_TotalEnergy | lh-entorhinal | 1 |
| HC_svPPA | gldm_GrayLevelNonUniformity | lh-entorhinal | 1 |
| HC_svPPA | gldm_SmallDependenceEmphasis | lh-entorhinal | 1 |
| HC_svPPA | glszm_ZonePercentage | lh-entorhinal | 1 |
| HC_svPPA | firstorder_Energy | Left-Amygdala | 2 |
| HC_svPPA | firstorder_TotalEnergy | Left-Amygdala | 1 |
| HC_svPPA | glrlm_GrayLevelNonUniformity | Left-Amygdala | 1 |
| svPPA_PNFA | glrlm_RunLengthNonUniformity | rh-temporalpole | 9 |
| svPPA_PNFA | gldm_DependenceNonUniformity | rh-temporalpole | 6 |
| svPPA_PNFA | firstorder_Energy | rh-temporalpole | 4 |
| svPPA_PNFA | firstorder_TotalEnergy | rh-temporalpole | 3 |
| svPPA_PNFA | gldm_GrayLevelNonUniformity | rh-temporalpole | 1 |
| svPPA_PNFA | glrlm_RunLengthNonUniformity | lh-temporalpole | 4 |
| svPPA_PNFA | gldm_DependenceNonUniformity | lh-temporalpole | 3 |
| svPPA_PNFA | firstorder_Energy | lh-temporalpole | 2 |
| svPPA_PNFA | firstorder_Skewness | lh-temporalpole | 2 |
| svPPA_PNFA | firstorder_TotalEnergy | lh-temporalpole | 2 |
| svPPA_PNFA | firstorder_Energy | lh-parahippocampal | 3 |
| svPPA_PNFA | firstorder_TotalEnergy | lh-parahippocampal | 2 |
| svPPA_PNFA | firstorder_TotalEnergy | lh-fusiform | 1 |
| svPPA_PNFA | firstorder_Energy | lh-entorhinal | 5 |
| svPPA_PNFA | firstorder_TotalEnergy | lh-entorhinal | 4 |
| svPPA_PNFA | glrlm_RunLengthNonUniformity | lh-entorhinal | 3 |
| svPPA_PNFA | gldm_DependenceEntropy | lh-entorhinal | 1 |
| svPPA_PNFA | glszm_LargeAreaLowGrayLevelEmphasis | Left-Amygdala | 1 |
